# A189 INFLAMMATORY BOWEL DISEASE IN PEDIATRIC PATIENTS POST RENAL TRANSPLANT:A CASE SERIES

**DOI:** 10.1093/jcag/gwab049.188

**Published:** 2022-02-21

**Authors:** A Sethuraman, O Guttman, C Barker, A Skinn

**Affiliations:** 1 Gastroenterology, BC Children’s Hospital, Vancouver, BC, Canada; 2 BC Children’s Hospital, Vancouver, BC, Canada

## Abstract

**Background:**

Diarrhea in renal transplant recipients may be due to infection, medication induced colitis and post-transplant lymphoproliferative disease. Uncommonly a subset of these children develop denovo inflammatory bowel disease (IBD), despite being already immunosuppressed. Paucity of literature in the pediatric population has been noted.

**Aims:**

To describe the clinical presentation, investigations and response to treatment in a cohort of children diagnosed with IBD post renal transplant

**Methods:**

Patients diagnosed with IBD post renal transplant between January 1 - October 1, 2021 at BC Children’s Hospital were identified. Medical history, symptoms at presentation, histological characteristics, results of investigations and response to treatment were reviewed

**Results:**

Data of 3 children were collected. Ages of the children were 11, 16 and 17 years (2 female). All patients had bloody diarrhea, abdominal pain and weight loss for 1 to 2 months. IBD diagnosis was made 8, 7 and 4 years respectively post renal transplant. All children were on maintenance immunosuppression with prednisone, MMF and tacrolimus. Bacterial, viral and parasitic causes were ruled out by relevant studies. All patients had negative anti-tissue transglutaminase.

Patient 1 (transplanted for renal dysplasia) was diagnosed with Crohn disease involving the antrum, ileum, and colon with rectal sparing. Fecal calprotectin (FC) was 1742 ug/g, CRP elevated at 56mg/L. Histology showed focally active chronic gastritis and changes in ileum with cryptitis and crypt dropout. The colon had features of moderate chronic active colitis with focal severe ulceration in the rectum. The patient achieved clinical remission on treatment with methylprednisolone, then started on vedolizumab in context of rising BK virus titre.

Patient 2 (transplanted for focal segmental glomerulosclerosis) was diagnosed with indeterminate colitis involving the IC valve and scattered colonic ulcers. FC was 674 ug/g and CRP and ESR were normal. Histology showed normal ileum, moderate active chronic changes in colon and rectal sparing. Clinical remission was achieved with oral mesalamine.

Patient 3 (transplanted for Bardet-Biedl syndrome) was diagnosed with indeterminate colitis with a colonoscopy that showed patchy colonic erythema and normal ileum. FC was 966 ug/g, and CRP and ESR were normal. Histology showed patchy chronic active colitis and mild active ileitis. Clinical remission was achieved with oral and rectal mesalamine.

**Conclusions:**

Denovo IBD occurs in children post renal transplant and must be considered in the differential for ongoing diarrhea in these patients. It can be a challenge to distinguish between IBD and other causes of diarrhea. The patients in this series entered clinical remission in a short period of time. Further exploration of the pathogenesis of IBD in these immunosuppressed patients would be valuable

**Funding Agencies:**

None

